# Surface Chemistry of Nanohybrids with Fumed Silica Functionalized by Polydimethylsiloxane/Dimethyl Carbonate Studied Using ^1^H, ^13^C, and ^29^Si Solid-State NMR Spectroscopy

**DOI:** 10.3390/molecules26195974

**Published:** 2021-10-01

**Authors:** Iryna S. Protsak, Yevhenii M. Morozov, Dong Zhang, Volodymyr M. Gun’ko

**Affiliations:** 1 Chuiko Institute of Surface Chemistry of National Academy of Sciences of Ukraine, 03164 Kyiv, Ukraine; iryna_protsak@yahoo.com; 2 BioSensor Technologies, AIT-Austrian Institute of Technology GmbH, 3430 Tulln, Austria; Yevhenii.Morozov@ait.ac.at; 3 Institute for Information Recording of National Academy of Sciences of Ukraine, 03113 Kyiv, Ukraine; 4 Department of Chemical & Biomolecular Engineering, University of Akron, Akron, OH 44325, USA; dz39@uakron.edu

**Keywords:** silica nanohybrids, solid-state NMR, surface properties, polydimethylsiloxane, silica functionalization

## Abstract

The investigation of molecular interactions between a silica surface and organic/inorganic polymers is crucial for deeper understanding of the dominant mechanisms of surface functionalization. In this work, attachment of various depolymerized polydimethylsiloxanes (PDMS) of different chain lengths, affected by dimethyl carbonate (DMC), to silica nanoparticles pretreated at different temperatures has been studied using ^29^Si, ^1^H, and ^13^C solid-state NMR spectroscopy. The results show that grafting of different modifier blends onto a preheated silica surface depends strongly on the specific surface area (SSA) linked to the silica nanoparticle size distributions affecting all textural characteristics. The pretreatment at 400 °C results in a greater degree of the modification of (*i*) A-150 (SSA = 150 m^2^/g) by PDMS-10/DMC and PDMS-1000/DMC blends; (*ii*) A-200 by PDMS-10/DMC and PDMS-100/DMC blends; and (*iii*) A-300 by PDMS-100/DMC and PDMS-1000/DMC blends. The spectral features observed using solid-state NMR spectroscopy suggest that the main surface products of the reactions of various depolymerized PDMS with pretreated nanosilica particles are the (CH_3_)_3_SiO-[(CH_3_)_2_SiO-]*_x_* fragments. The reactions occur with the siloxane bond breakage by DMC and replacing surface hydroxyls. Changes in the chemical shifts and line widths, as shown by solid-state NMR, provide novel information on the whole structure of functionalized nanosilica particles. This study highlights the major role of solid-state NMR spectroscopy for comprehensive characterization of functionalized solid surfaces.

## 1. Introduction

Nanooxides such as fumed silica (nanosilica) are important industrial materials used in both unmodified [1,2,3,4,5] and modified functionalized [6,7,8,9,10,11,12,13] states. Various silanes and siloxanes are widely used to modify silica particles [14,15,16,17,18,19,20,21,22]. Modified silica materials with alkyl chains are more appropriate to prepare various composites with polymers as compared with unmodified silica materials [23,24,25,26,27,28,29,30,31,32]. Hybrid organic(organometallic)–silica materials are an important class of composites of high interest ranging from fundamental developments to advanced applications. In composites, organic (or organometallic) fragments (functional groups) are covalently bound to a matrix [5,6,7,8,33,34,35,36,37,38,39,40,41]. Organophilization of a silica surface can be performed using different kinds of modifying agents, including organosiloxanes as the most attractive and environmentally friendly silylating reagents [5,6,7,8,9,10,11,12]. Linear organosiloxanes are generally not considered to be reactive with inorganic oxide surfaces, and enormous efforts have been made over the last 50 years to develop silicon-containing reagents with reactive functional groups [42]. Previously, it was found that dimethyl carbonate (DMC) [43], which is an environmentally friendly reagent [44,45,46], can promote partial depolymerization of organosiloxanes (e.g., polydimethylsiloxane, PDMS) and breakage of the siloxane bonds in a surface layer of silica nanoparticles. These reactions allow the silica surface hydrophobization by PDMS fragments under relatively mild conditions. Thus, PDMS/DMC mixtures may be an excellent candidate for silica surface hydrophobization.

Understanding the performance of nanosilica functionalization requires a molecular-level characterization of the functional groups, including their conformations and modes of binding/interaction to/with a surface [35]. Different bonding schemes of attachment of the polymer fragments to a silica surface may lead to different geometry of a modified surface layer. Knowledge on the surface incorporation patterns and conformations of functional groups is an important step toward developing smart functional substrates. Nuclear magnetic resonance (NMR) spectroscopy is one of the most suitable methods to study the above-mentioned features of initial and modified surface layers [8,47,48,49,50,51,52,53,54].

The particulate morphology and texture of the fumed nanosilica powders depend strongly on several factors: (i) specific surface area (SSA, S_BET_), particle size distributions (PaSD), and textural pore size distributions (PSD); (ii) pre-treatment history (heating, wetting-drying, mechanical loading, aging, etc.); and (iii) time of treatments [5,6,7,8,9,48,55,56,57,58]. Many of these changes are due to hydration–dehydration of a surface and volume of silica nanoparticles during storage and pretreatments of nanosilica particles. Therefore, not only SSA, PaSD, and PSD, but also preheating conditions of nanosilica particles should be taken into account during silica surface functionalization. Thus, the aim of the present study was to thoroughly investigate features of surface functionalization of various nanosilica particles (A-150, A-200, and A-300), characterized by different SSA, PaSD, and PSD and pretreated at different temperatures (200, 400, and 600 °C) by various PDMS/DMC blends using solid-state NMR spectroscopy.

## 2. Experimental Methods

### 2.1. Chemical Reagents and Nanosilica Particle Characterization

**Scheme 1:**
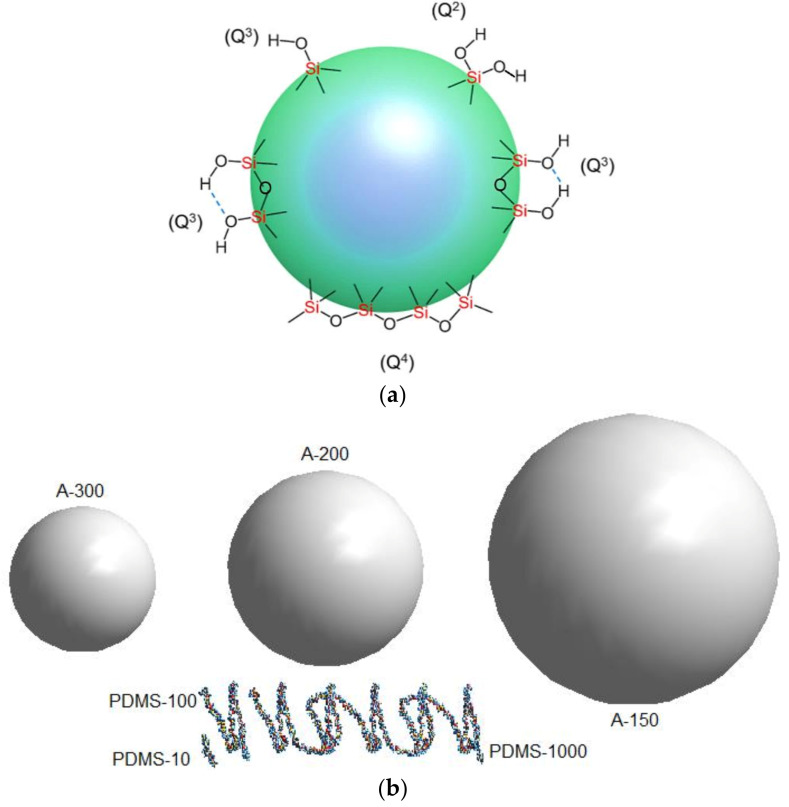
(**a**) Types of silanol groups and siloxane bridges at a surface of amorphous silica particles: twin (Q^2^), and isolated (Q^3^) silanols, Q^4^ denotes Si with four -O-Si bonds; (**b**) scales of NPNP of silica particles and PDMS of the molecular weights of ca. 1.25, 6, and 30 kDa and the degree of polymerization of 15, 80, and 400, respectively.

To modify three nanosilica samples, A-300 (S_BET_ ≈ 300 m^2^/g, average diameter of nanoparticles *d* = 9.1 nm, Scheme 1b), A-200 (200 m^2^/g, *d* = 13.6 nm), and A-150 (150 m^2^/g, *d* = 18.2 nm) (Aladdin Reagents, Shanghai, China), three polydimethylsiloxanes (linear and -CH_3_ terminated (CH_3_)_3_SiO[Si(CH_3_)_2_O]_n_Si(CH_3_)_3_): PDMS-10 (viscosity of ca. 10 cSt at 25 °C, molecular weight ~1250 Da, degree of polymerization *n* ≈ 15, Scheme 1b); PDMS-100 (100 cSt at 25 °C, molecular weight ~6 kDa, degree of polymerization ~80), and PDMS-1000 (1000 cSt at 25 °C, molecular weight ~30 kDa, degree of polymerization ~400) (Aladdin Reagents, Shanghai, China) have been used with dimethyl carbonate (DMC, (CH_3_O)_2_CO) as a siloxane bond breakage agent. The reagents have been used as received.

**Figure 1:**
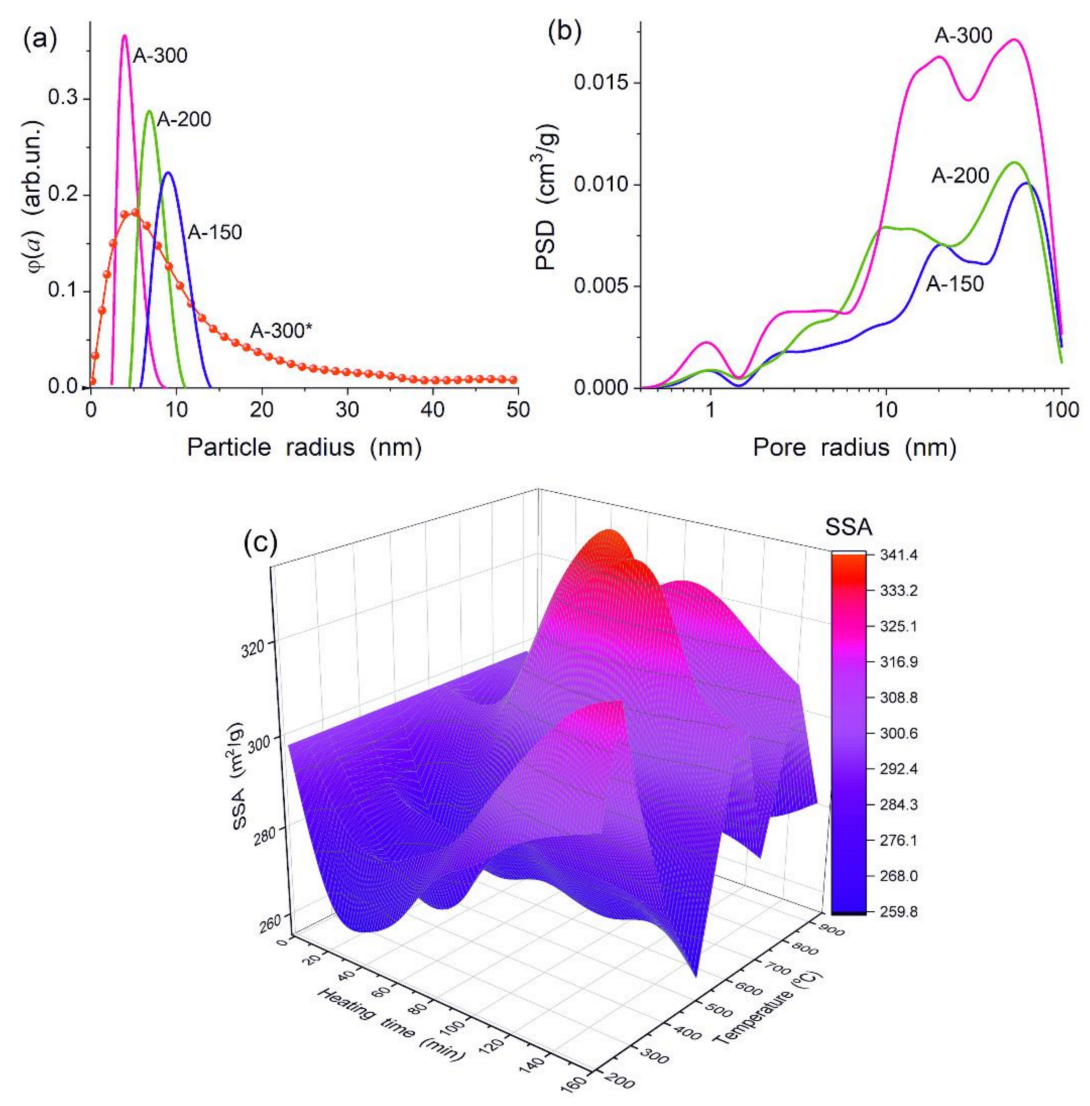
(**a**) Particle size distributions (PaSD) for fumed silica calculated using the nitrogen adsorption isotherms and A-300 PaSD (A-300*) calculated using the small angle X-ray scattering (SAXS [58]) data; (**b**) incremental pore size distributions (calculated nitrogen isotherms with a model of voids between NPNP [59]); and (**c**) specific surface area (using low-temperature Ar adsorption [48]) vs. time and temperature of heating of A-300.

Nonporous primary nanoparticles (NPNP) of fumed silica are characterized by broader PaSD with increasing NPNP sizes (decreasing SSA) (Figure 1a). However, the SAXS PaSD (computed with a model of spherical particles) for A-300 is much broader (Figure 1a, A-300*) than the PaSD (A-300) computed from the nitrogen adsorption data using a self-consistent regularization procedure [48]. This result is explained by NPNP aggregation, since SAXS data for several neighboring particles in aggregates could be interpreted as for one larger particle. Thus, a long tail in the SAXS PaSD for A-300 corresponds to aggregates of NPNP observed in microscopic images [48]. The NPNP aggregation and the formation of agglomerates of aggregates may play an important role upon the silica surface modification by polymers (PDMS) of different lengths (different molecular weight).

Voids between NPNP in their aggregates and agglomerates of aggregates provide large empty volume (*V*_em_) in the powders *V*_em_ = 1/ρ_b_–1/ρ_0_, where ρ_b_ and ρ_0_ are the bulk (0.04–0.13 g/cm^3^) and true density (2.2 g/cm^3^) of nanosilica particles that gives the *V*_em_ range of 24.5–7.2 cm^3^/g. Upon the nitrogen adsorption, only a small part (<10%) of *V*_em_ is filled by the N_2_ molecules at relative pressures *p*/*p*_0_ > 0.98 because of extremely weak interactions between adsorbate molecules and distant NPNP in macrovoids in the agglomerates. Nitrogen mainly fills nano/mesovoids and only partially fills macrovoids independent of the SSA values (or PSD) of nanosilica particles (Figure 1b) and pretreatment history of samples, e.g., mechanical or hydrocompaction of fumed silica [48] or heating (Figure 1c).

Pretreatment conditions, e.g., temperature, can affect the SSA value. This is clearly seen from 3D *S-T-t* dependence for A-300 (Figure 1c), estimated using the Ar adsorption isotherms to avoid the adsorbate molecule orientation effects characteristic for nitrogen molecules [48]. These changes are due to several processes caused by dehydration of silica nanoparticles. Intact water bound to a surface of NPNP desorbed mainly at *T* < 150 °C and samples preheated at 200 °C for several hours (typical pretreatment of silica particles before the adsorption measurements) do not practically include intact water at a surface. However, there is water released from the nanoparticle volume upon heating. This water origin is linked to both hydroxyls and intact intraparticle-bound molecules. Note that a small fraction of isolated hydroxyls can remain at a silica surface during treatment even at 1000 °C due to large distances between residual silanols for the condensation reaction. Upon strong dehydration, NPNP sintering could occur (resulting in decreasing SSA), but NPNP dehydration per se causes an increase in the SSA value. Additionally, all of these processes depend on synthesis features and sample prehistory. Therefore, the 3D picture of SSA vs. *T* and heating time (*t*) is relatively complex (Figure 1c). The particulate morphology and texture of nanosilica particles (Figure 1) play an important role upon functionalization of a surface of NPNP because they provide appropriate surface accessibility for such polymers (or fragments) as PDMS.

### 2.2. Functionalization of Nanosilica Particles

Three PDMS samples (Table 1) were chosen as environmentally benign modifying reagents with a high carbon content. Three samples of fumed silica, applied as a matrix for modification, are characterized by different particulate morphology and texture (Figure 1). The silica samples were pretreated at 200, 400, or 600 °C for 2 h (Table 1). The PDMS/DMC (1:1) blends (added by means of aerosol-nozzle spray) reacted with silica samples at 200–220 °C for 2 h. The amount of a modifier agent (PDMS) was 17 wt% with respect to the silica weight in all cases (that is greater than the amounts needed to provide monomolecular coverage of the studied silica particles). The functionalization process was performed in a glass reactor with a stirrer (350–500 rpm). After the modification reactions, the samples were cooled to room temperature. Silica functionalization with PDMS/DMC corresponds to several reactions with the cleavage of the siloxane bonds (1) and (2) and subsequent attachment of the PDMS fragments (3) and (4):

(CH_3_)_3_SiO-[Si(CH_3_)_2_-O]_n_-Si(CH_3_)_3_ + (CH_3_-O)_2_CO→(CH_3_)_3_SiO-[Si(CH_3_)_2_-O]_x_-CH_3_ + (CH_3_)_3_SiO-[Si(CH_3_)_2_-O]_y_-CH_3_ + CO_2_ (1)

≡Si-O-Si≡ + CH_3_-O-C(O)-OCH_3_ → 2(≡Si-O-CH_3_) + CO (2)

≡Si-OH + (CH_3_)_3_Si-O-[Si(CH_3_)_2_-O]_x_-CH_3_→≡Si-O-[Si(CH_3_)_2_-O]_x_-Si(CH_3_)_3_ + CH_3_OH (3)

≡Si-O-CH_3_ + (CH_3_)_3_Si-O-[Si(CH_3_)_2_-O]_x_-CH_3_→≡Si-O-[Si(CH_3_)_2_-O]_x_-Si(CH_3_)_3_ + CH_3_OCH_3_ (4)

### 2.3. ^29^Si, ^1^H and ^13^C CP/MAS NMR Spectroscopy

Solid-state (magic-angle spinning, MAS) ^1^H NMR spectra were recorded using a Bruker Avance 400 III HD spectrometer (Bruker, San Jose, USA, magnetic field strength 9.3947 T) at a resonance frequency of 400.15 MHz. The powder samples were placed in a pencil-type zirconia rotor of 4.0 mm o.d. The spectra were recorded at a spinning speed of 10 kHz, with a recycle delay of 1 s. The adamantane was used as the reference for estimation of the chemical shift of the proton resonance.

Solid-state ^29^Si cross-polarization (CP)/MAS NMR spectra were recorded (using the same spectrometer) at a resonance frequency of 79.49 MHz using the cross-polarization, magic-angle spinning, and high-power ^1^H decoupling. The powder samples were placed in a pencil-type zirconia rotor of 4.0 mm o.d. The spectra were obtained at a spinning speed of 8 kHz (4 µs 90° pulses), an 8 ms CP pulse, and a recycle delay of 4 s. The Si signal of tetramethylsilane (TMS) at 0 ppm was used as the reference of ^29^Si chemical shift.

Solid-state ^13^C CP/MAS NMR spectra were recorded at a resonance frequency of 100.62 MHz for ^13^C and a high-power ^1^H decoupling. The powder samples were placed in a pencil-type zirconia rotor of 4.0 mm o.d. The spectra were obtained at a spinning speed of 5 kHz (4 µs 90° pulses), a 2 ms CP pulse, and a recycle delay of 4 s. The methylene signal of glycine at 176.03 ppm was used as the reference of ^13^C chemical shift.

## 3. Results and Discussion

**Figure 2:**
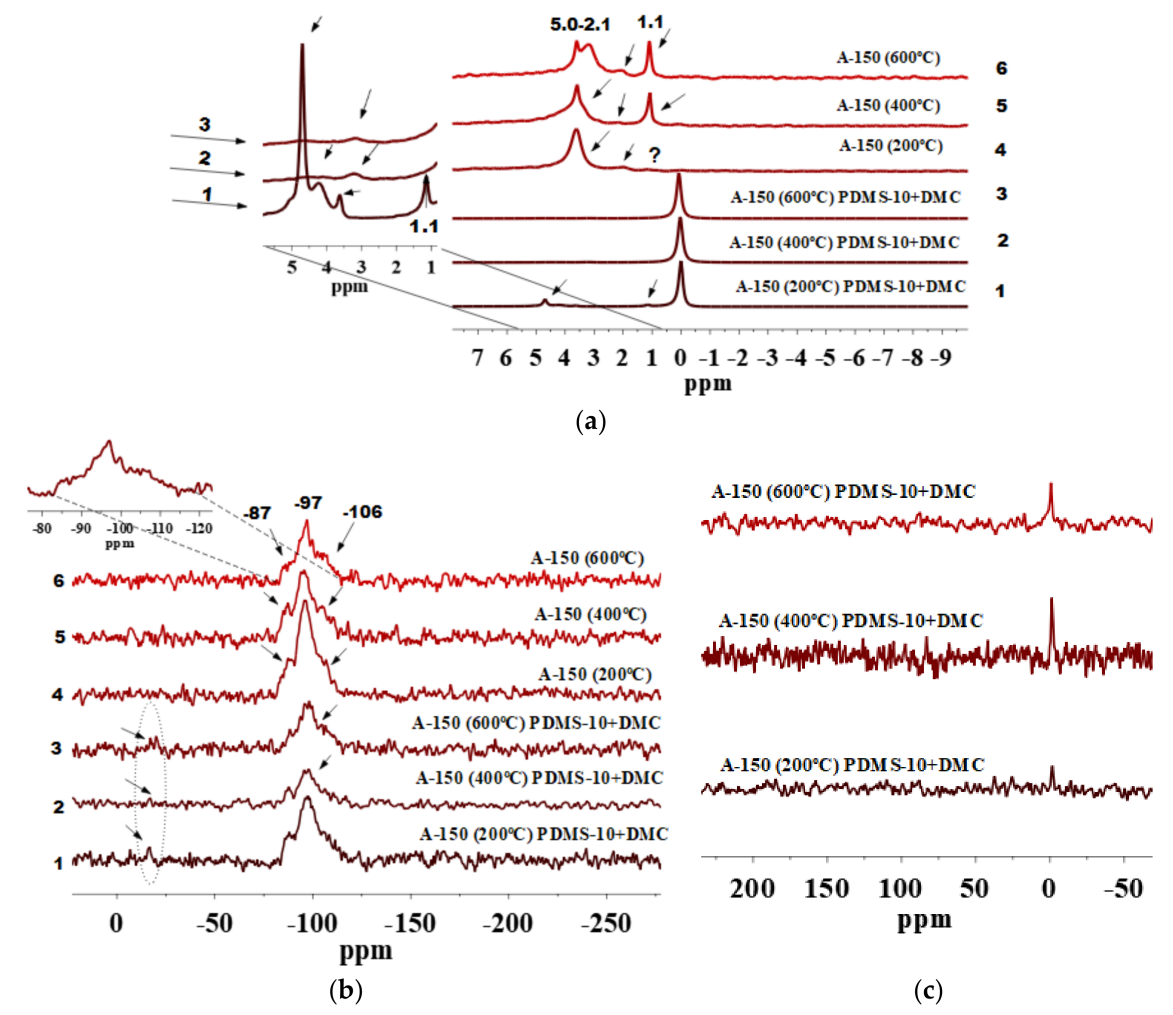
(**a**) ^1^H MAS NMR, (**b**) ^29^Si CP/MAS NMR and (**c**) ^13^C CP/MAS NMR spectra of fumed silica A-150 preheated at 200, 400 and 600 °C and modified by PDMS-10/DMC.

^1^H MAS NMR spectra of unmodified fumed silica preheated at different temperatures (200 °C, 400 °C, and 600 °C, Figure 2a, lines 4–6) represent two main peaks of the chemical shifts of the proton resonance at δ_H_ = 1.1 and 2.1–5.0 ppm. The peaks in the range of 2.1–5.0 ppm are related to twin (Q^2^) and vicinal silanols (Q^3^), disturbed single silanols (Q^3^) (Scheme 1), as well as intraparticle-bound water (IPBW) molecules [48]. The peak at 1.1 ppm could be assigned to isolated silanols non-interacting with neighbors or bound water molecules at a silica surface. It is known that protons in isolated silanols could give signals at δ_H_ = 0.5–1.5 ppm. Similar spectral features were previously observed [48,50]. Single ≡SiOH groups are called as isolated silanols if the distances to the closest neighbor ≡SiOH groups are longer than that characteristic for the hydrogen bonds. Therefore, silanols separated by more than ~0.33 nm can be considered as isolated [5,6,7,8,51]. Pairs of silanols belonging to tetrahedra that share a common oxygen atom are called as vicinal silanols (Scheme 1). Two OH groups linked to the same surface silicon atom give twin =Si(OH)_2_ moiety. These OH are close located but oriented in such a way that they cannot be involved into strong hydrogen bond [48,51]. The weaker the hydrogen bond, the smaller is the δ_H_ value. In other words, the hydrogen bond between vicinal silanols is stronger (δ_H_ = 4–5 ppm) than that for twin silanols (δ_H_ = 2–3 ppm). In liquid bulk or adsorbed water (δ_H_ = 4.0–5.5 ppm) with mobile molecules, the hydroxyl orientation is less appropriate to form the strong hydrogen bonds as it is observed in Ih (hexagonal ice with δ_H_ = 7.0 ppm) [48].

Signal at 1.1 ppm is observed for a pure A-150 preheated at 400 and 600 °C (Figure 2a, lines 5 and 6) but it is hardly detectable for A-150 preheated at 200 °C (Figure 2a, line 4). This can be explained by the fact that after preheating at 200 °C, there are larger amounts of strongly bound water and various silanols [8,48] than after preheating at 400 or 600 °C. Therefore, isolated silanols appear in a larger amount after preheating at 400 or 600 °C because the amounts of twin and vicinal silanols and IPBW molecules decrease.

Beside various silanols which remained at the surface of silica particles pretreated at 400 and 600 °C, as shown in the ^1^H MAS NMR spectra, water molecules can be retained inside nanoparticles and appear at the range of δ_H_ = 3–5 ppm (Figure 2a, lines 4–6). Nevertheless, the signals decrease after the modification (Figure 2a, lines 1–3). Therefore, they are linked to various silanols rather than IPBW. However, they do not disappear completely because some of the OH groups (e.g., located in narrow voids between adjacent NPNP) and IPBW are inaccessible for the modifier reagents. The contents of various silanols inaccessible for a modifier were estimated by Marciel et al. [52]. For samples evacuated at 200, 400, and 550 °C, this was about 45, 15, and 8% of the total amount of the OH groups, respectively. This correlates with our results. Figure 2a, (lines 4–6) shows that by increasing the preheating temperature to 400 and 600 °C, the contribution of isolated silanols grows in comparison with silica samples pretreated at 200 °C (Figure 2a, line 4). During dehydration at different temperatures, contributions of various silanols can differently change with temperature because of different locations and surroundings [48,60,61]. An extremely small amount of residual silanols can remain upon heating even at 1000 °C.

The ^1^H MAS NMR spectra of A-150 preheated at 200, 400, and 600 °C and modified with the mixture of PDMS-10/DMC include signal of ≡Si-CH_3_ at δ_H_ = 0 ppm. This confirms the attachment of PDMS fragments to the silica surface (Figure 2a, lines 1–3). Notice that modified A-150 samples pretreated at 400 and 600 °C (Figure 2a, lines 2 and 3) show decreased signals at δ_H_ = 1.1–5.0 ppm as compared with the unmodified A-150 which was preheated (Figure 2a, lines 5 and 6). This confirms a significant degree of the silica functionalization. However, A-150 pretreated at 200 °C and modified (Figure 2a, line 1) shows the presence of all types of silanols and bound water as compared with unmodified silica particles (Figure 2a, line 4).

The ^29^Si CP/MAS NMR spectra of unmodified A-150 pretreated at different temperatures (Figure 2b) show three signals with resolved peaks at −87 to −91, −97 to −101, and −106 to −110 ppm. These peaks are assigned to silicon atoms in twin and single silanols and silicon-oxygen tetrahedra in the silica framework, respectively: Q^2^, Q^3^, and Q^4^, where the superscript indicates a number of the siloxane bonds [54].

As it can be seen from Figure 2b, lines 1–3, after grafting of PDMS-10/DMC at the pretreated A-150 surface (200, 400, and 600 °C), a significant decrease in the signals Q^2^ and Q^3^ occurs as compared with unmodified silica particles (Figure 2b, lines 4–6). This is accompanied by the appearance of a low intensity peak at −15 to −17 ppm. It can be assigned to dimethylsiloxane units abbreviated as D^2^, having the structure of -[-OSi(CH_3_)_2_)-]_x_ related to grafted PDMS fragments [37,48]. The intensity of D^2^ peaks is not high, but the differences in intensity of Q^2^ and Q^3^ before (Figure 2b, lines 4–6) and after modification (Figure 2b, lines 1–3) are clearly visible. The intensity of the Q^2^ and Q^3^ peaks is reduced as compared with the unmodified silica particles. The grafting of PDMS species is confirmed by the ^13^C CP/MAS NMR spectra (Figure 2c) with a peak at −1.5 ppm. From the spectra mentioned above, it is apparent that grafting of (CH_3_)_3_SiO[-(CH_3_)_2_SiO-]*_x_* groups onto heat-pretreated silica surface produces species forming homogenous local structures since the D^2^ signal remains relatively narrow [62]. Higher intensity of a signal at −1.5 ppm in ^13^C CP/MAS NMR for modified silica particles pretreated at 400 °C and 600 °C (Figure 2c) may suggest a greater number of attached surface species as compared with A-150 pretreated at 200 °C. Notice that intensities of the ^29^Si signals do not match with the intensities of the ^13^C NMR signals. This is obvious since peak intensity depends greatly on such factors as natural abundance of the nuclei, sensitivity of the probe, experimental conditions applied during each measurement, etc. [48,63,64,65]. To this point, ^29^Si has an inherently low natural abundance (4.7%) which requires extended acquisition time to obtain spectra with an acceptable sensitivity [64].

**Figure 3:**
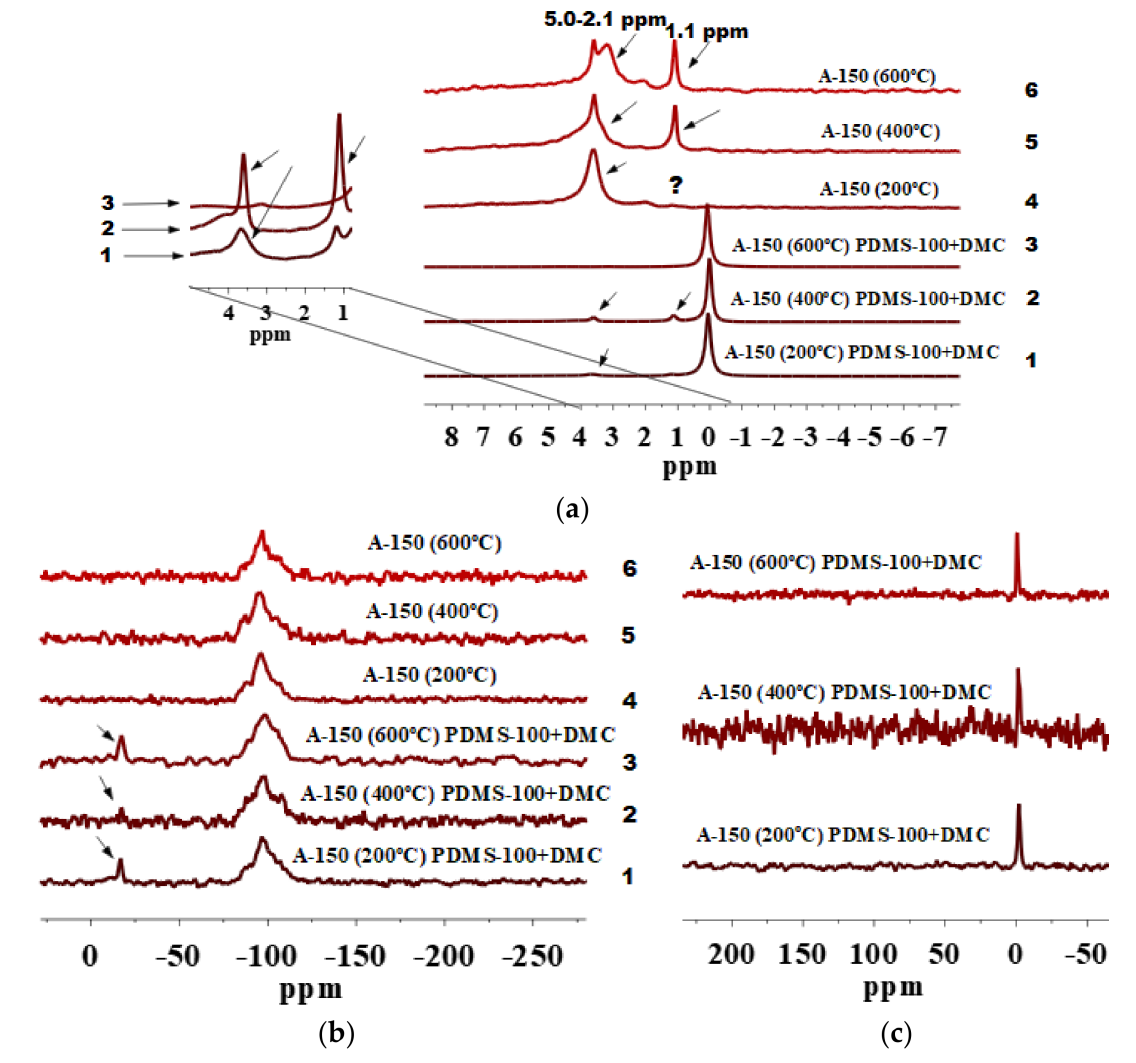
(**a**) ^1^H MAS NMR, (**b**) ^29^Si CP/MAS NMR and (**c**) ^13^C CP/MAS NMR spectra of fumed silica A-150 preheated at 200, 400 and 600 °C and modified by PDMS-100/DMC.

The ^1^H MAS NMR spectrum of functionalized A-150 pretreated at 600 °C (Figure 3a, line 3) shows full involvement of residual silanols in the reactions with PDMS-100/DMC, since peaks located in the range of 1.1–5.0 ppm are not visible compared with unmodified silica samples pretreated at 600 °C (Figure 3a, line 6). It could be explained by the fact that after silica heating at 600 °C, silanols were not completely removed from the surface (Figure 3a, line 6). However, this is for mainly isolated silanols, which take part in the reactions with PDMS/DMC.

Notice that the ^1^H MAS NMR spectra of modified A-150 pretreated at 200 and 400 °C show residual peaks of all silanols and IPBW (Figure 3a, lines 1 and 2) at 1.1–5.0 ppm. This reveals partial participation of these active sites in the reactions with the modifiers as compared with the initial silica particles (Figure 3(a), lines 4 and 5). The grafting of alkyl species at these silica particles can be confirmed by the appearance of the shift at about 0.0 ppm in ^1^H MAS NMR spectra (Si-CH_3_) (Figure 3a, lines 1–3), shifts from −15 to −17 ppm (Figure 3b, lines 1–3) in ^29^Si CP/MAS NMR, and a shift at about −1.5 ppm in the ^13^C CP/MAS NMR spectra (Figure 3c). Notice that D^2^ sites are with almost no changes in signals vs. preheating temperature of silica particles (Figure 3b, lines 4–6). Similar results were recorded for functionalized SBA-15 [35] investigated using MAS combining with dynamic nuclear polarization (DNP). It was found that the signals of the Q^n^ sites build up progressively with increasing cross polarization contact time τ_CP_ and transferring polarization from more distant spins. Additionally, a lower sensitivity of ^29^Si CP/MAS NMR could be explained rather by the properties of silicon atoms than by the hydroxy environment of surface silanols. Therefore, changes in the intensity of these peaks are not always clearly visible.

**Figure 4:**
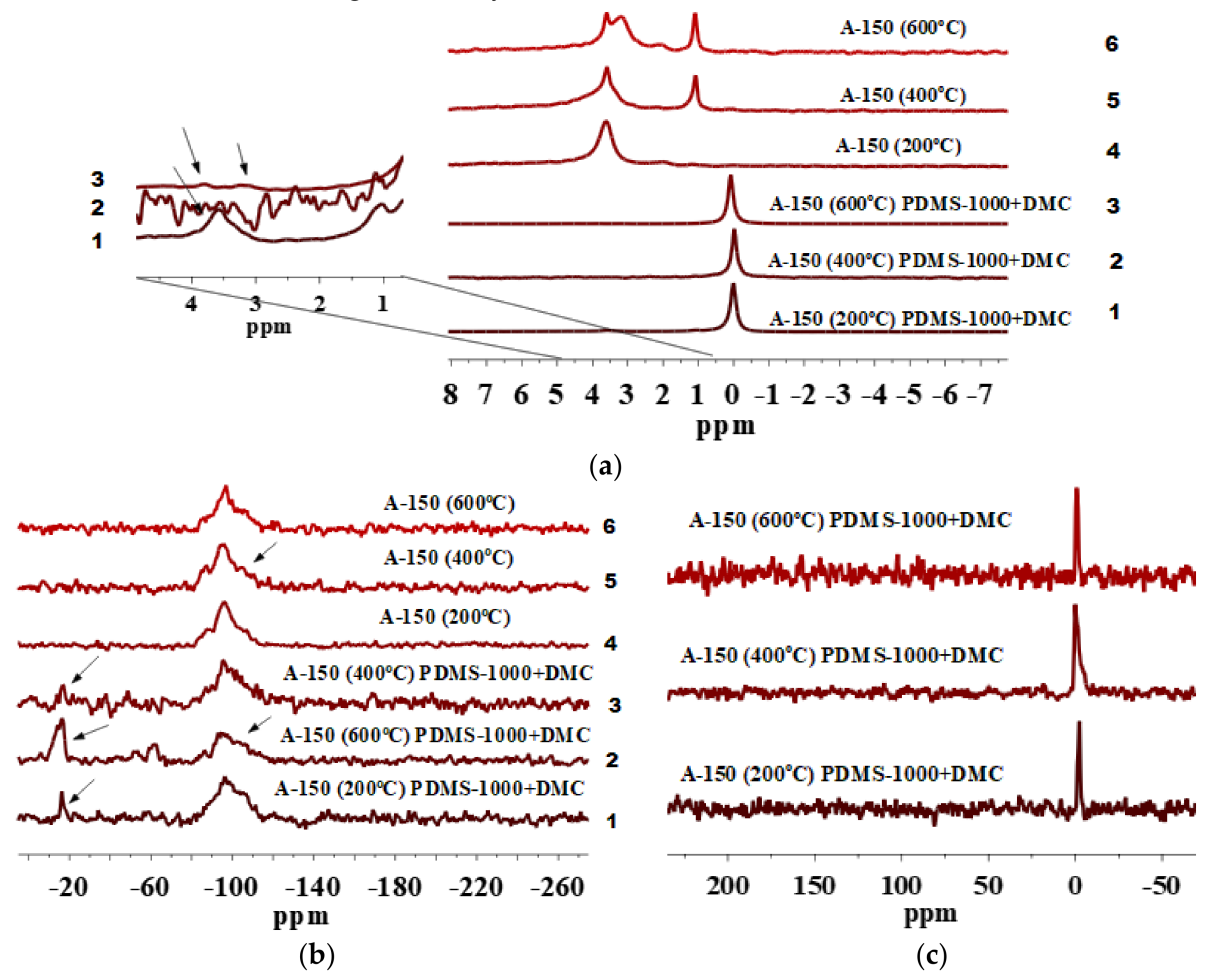
(**a**) ^1^H MAS NMR, (**b**) ^29^Si CP/MAS NMR, and (**c**) ^13^C CP/MAS NMR spectra of A-150 preheated at 200, 400, and 600 °C and modified by PDMS-1000/DMC.

The ^1^H MAS NMR spectra of pretreated A-150 at 400 and 600 °C and modified with the longest PDMS-1000 in the presence of DMC (Figure 4a, lines 2 and 3) show full participation of all residual hydroxyls with the modifier mixture. The peaks corresponding to these groups at 1.1–5.0 ppm are hardly detectable compared with the unmodified silica particles (Figure 4a, lines 5 and 6).

Notice that A-150 pretreated at 200 °C and modified with PDMS-1000/DMC is characterized by the presence of residual hydroxyls and IPBW (Figure 4a, line 1) as is shown above for A-150/PDMS-10/DMC (Figure 2a, line 1) and A-150/PDMS-100/DMC (Figure 3a, line 1). This can be explained by the fact that preheating of A-150 at 200 °C leads to the removal of a small quantity of silanols and IPBW. Even after silica modification, a fraction of these sites remains. Nevertheless, modified A-150, similarly pretreated (Figure 4a-c, lines 1–3), shows grafted PDMS fragments that are confirmed by the appearance of a peak at 0.0 ppm in the ^1^H MAS NMR spectra (Figure 4a, lines 1–3). Additionally, the ^29^Si and ^13^C CP/MAS NMR spectra show signals of grafted PDMS fragments for all modified samples because signals of D^2^ and carbon atoms in dimethylsilyl groups appear (Figure 4b, lines 1–3 and Figure 4c). Notice that the ^13^C CP/MAS NMR spectrum of A-150 pretreated at 400 °C and modified with PDMS-1000/DMC shows a line broadening effect. This could reveal different orientations of closely adjacent surface functionalities under steric effects.

According to the ^29^Si CP/MAS NMR spectrum of A-150 pretreated at 400 °C and modified with PDMS-1000/DMC (Figure 4b, line 2), the highest amounts of grafted groups are observed since peak D^2^ is higher than that for other silica samples (Figure 4b, lines 1 and 3). Additionally, growing D^2^ is accompanied by a visible decrease in Q^3^ and Q^2^ as compared with the unmodified silica particles (Figure 4b, line 5). The appearance of only the D^2^ peak related to PDMS in ^29^Si CP/MAS NMR suggests that the major surface products of the reactions of PDMS/DMC with the silica surface correspond to the PDMS fragments only.

Comparison of A-150 modified by three PDMS/DMC blends reveals that the use of PDMS-1000/DMC leads to a relatively higher degree of modification (see ^29^Si and ^13^C NMR in Figure 2, Figure 3, Figure 4b; Figure 4c) compared with that of samples modified with PDMS-100/DMC and PDMS-10/DMC. Additionally, A-150 pretreated at 400 °C and modified with PDMS-1000/DMC demonstrates a relatively higher grafting degree in comparison with silica samples pretreated at 200 and 600 °C and modified with PDMS-1000/DMC. These features should be compared with ones observed for A-200 and A-300 preheated and functionalized in similar ways as A-150.

**Figure 5:**
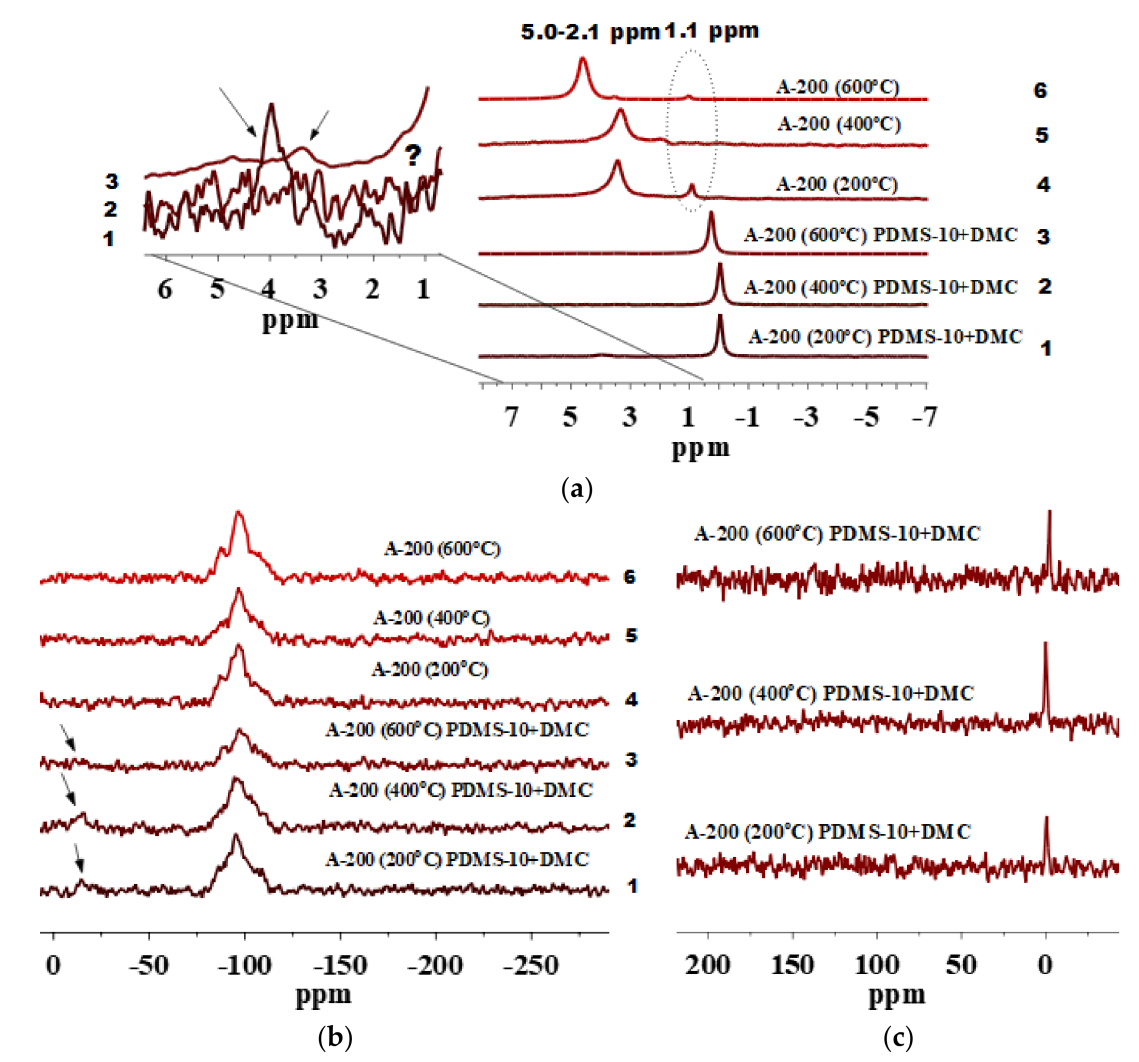
(**a**) ^1^H MAS NMR, (**b**) ^29^Si CP/MAS NMR, and (**c**) ^13^C CP/MAS NMR spectra of fumed silica A-200 preheated at 200, 400, and 600 °C and modified by PDMS-10/DMC.

For A-200, isolated silanols at unmodified silica samples pretreated at different temperatures (Figure 5a, lines 4–6) are hardly detectable in the ^1^H MAS NMR spectra. This may be explained by the morphological and textural features of A-200 (Figure 1). The smaller the NPNP, the stronger is their aggregation [48]. Additionally, A-200 contains more twin and vicinal silanols than A-150 (Figure 2a, Figure 3a and Figure 4a, lines 4–6). The ^1^H MAS NMR spectra of unmodified preheated A-200 have peaks at 2.1–5.0 ppm (Figure 5a, lines 4–6) confirming the presence of various silanols (Scheme 1) and IPBW molecules. After modification of preheated A-200 with PDMS-10/DMC, the silanol amount decreases (Figure 5a, lines 1–3). This is accompanied by the appearance of grafted PDMS fragments giving δ_H_ = 0.0 ppm. The grafting of the functionalities is also confirmed by the appearance of the D^2^ peak in the ^29^Si CP/MAS NMR spectra, as well as a peak at −1.5 ppm in the ^13^C CP/MAS NMR spectra (Figure 5b, lines 1–3, and Figure 5c). Note that A-200 pretreated at 400 °C and modified with PDMS-10/DMC (Figure 5b, line 2, and Figure 5c) is characterized by the highest amount of dimethylsilyl groups since relative intensity of the corresponding species are higher as compared with other silica particles.

**Figure 6:**
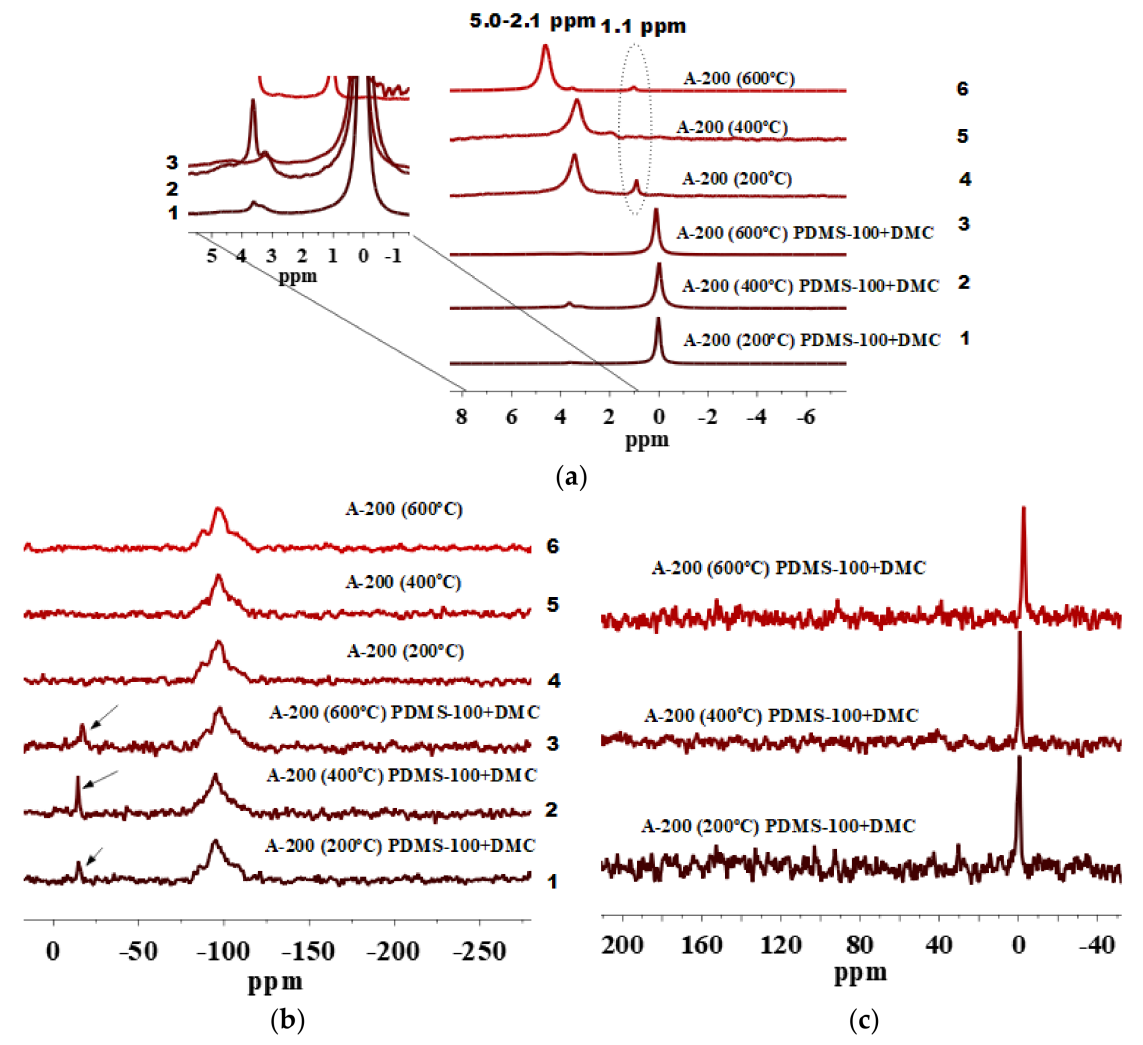
(**a**) ^1^H MAS NMR, (**b**) ^29^Si CP/MAS NMR, and (**c**) ^13^C CP/MAS NMR spectra of A-200 preheated at 200, 400, and 600 °C and modified by PDMS-100/DMC.

As it can be seen from the ^1^H MAS NMR spectra of unmodified A-200 pretreated at different temperatures (Figure 6a, lines 4–6) and then modified with the PDMS-100/DMC mixture (Figure 6a, lines 1–3), signals of isolated silanols at 1.1 ppm are hardly visible. However, it is visible for the same silica sample but modified with PDMS-10/DMC (Figure 5a, lines 4–6). This confirms the fact that amounts of twin and vicinal silanols are high for this silica sample. This could lead to certain shielding effects for isolated silanols of low intensity (Figure 6a).

Besides low intensity peaks of isolated silanols (Figure 6a, lines 4–6), the ^1^H MAS NMR spectra have peaks of twin and vicinal silanols at 2.1–5.0 ppm and peaks corresponding to the grafted PDMS species at 0.0 ppm. The anchoring of these functionalities is confirmed by D^2^ signals in the ^29^Si CP/MAS NMR spectra (Figure 6b, lines 1–3), and signals at −1.5 ppm in the ^13^C CP/MAS NMR spectra (Figure 6c).

Note that silica A-200 samples pretreated at 400 °C and then modified by PDMS/DMC is characterized by the highest amount of dimethylsilyl groups as it is clear from the ^29^Si CP/MAS NMR spectrum (Figure 6b, line 2). The same is also observed for A-200 modified by PDMS-10/DMC (Figure 5b, line 2).

**Figure 7:**
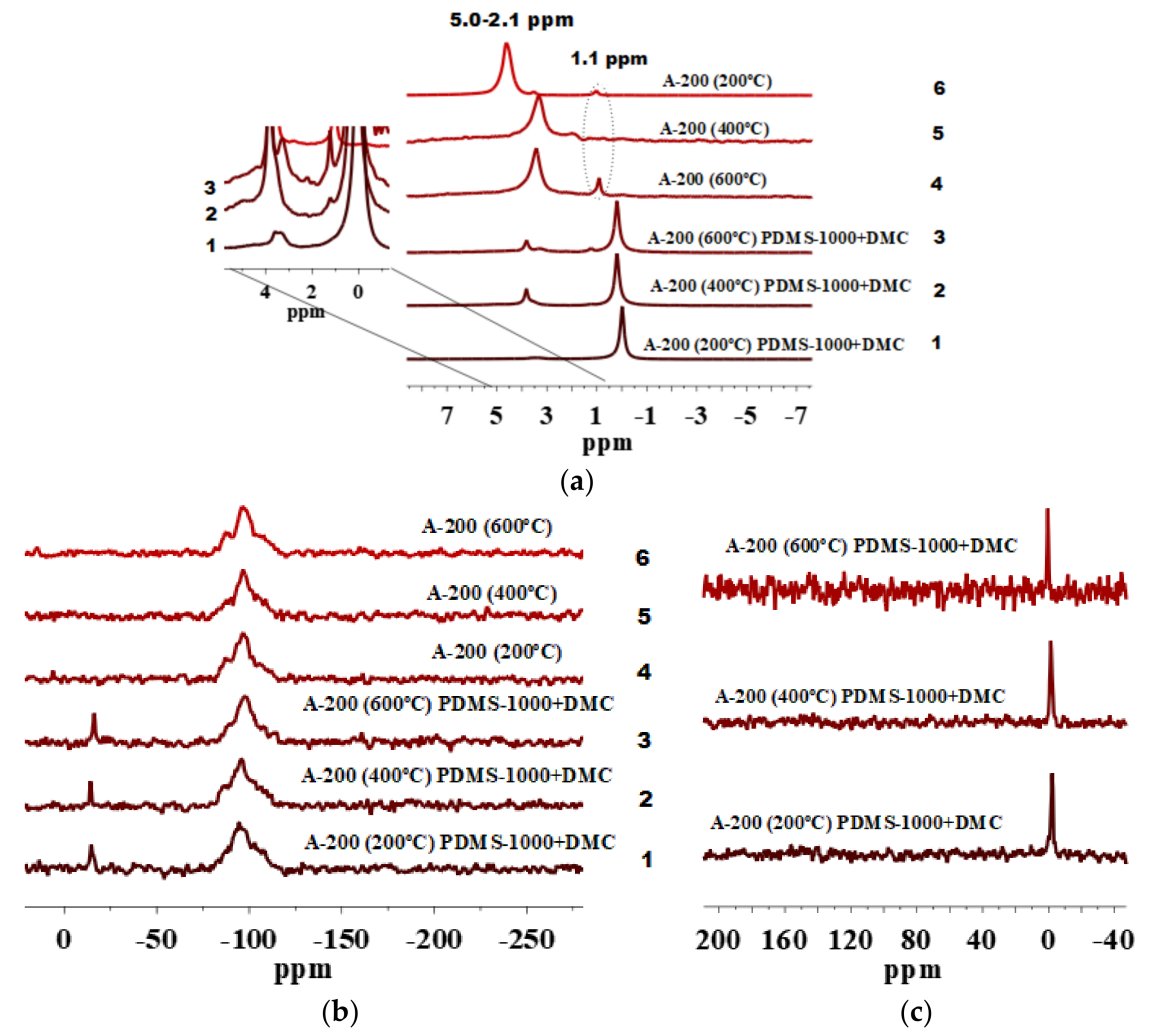
(**a**) ^1^H MAS NMR, (**b**) ^29^Si NMR, and (**c**) ^13^C CP/MAS NMR spectra of A-200 preheated at 200, 400, and 600 °C and modified by PDMS-1000/DMC.

The ^29^Si and ^13^C CP/MAS NMR spectra of silica A-200 samples modified by long PDMS-1000/DMC (Figure 7b,c) are characterized by practically the same intensity D^2^ peak (Figure 7b, lines 1–3) and a peak at −1.5 ppm (Figure 7c), respectively. This shows a similar degree of PDMS fragments at a silica surface showing a ^1^H MAS peak at 0.0 ppm (Figure 7a, lines 1–3). Thus, grafting of the functionalities depends not only on the SSA value and pretreatment temperature, but also on the length of the PDMS chain (PDMS-10, PDMS-100, PDMS-1000). However, the increase in the PDMS length does not lead to a higher degree of PDMS grafting onto A-200 (Figure 5, Figure 6 and Figure 7a–c), since the highest degree of PDMS grafting onto A-200 is observed for PDMS-100/DMC (Figure 6b,c). The silica modification studied is performed in the presence of DMC, but the difference in the DMC reactions with PDMS characterized by different molecular weights remains unclear and needs additional investigations.

**Figure 8:**
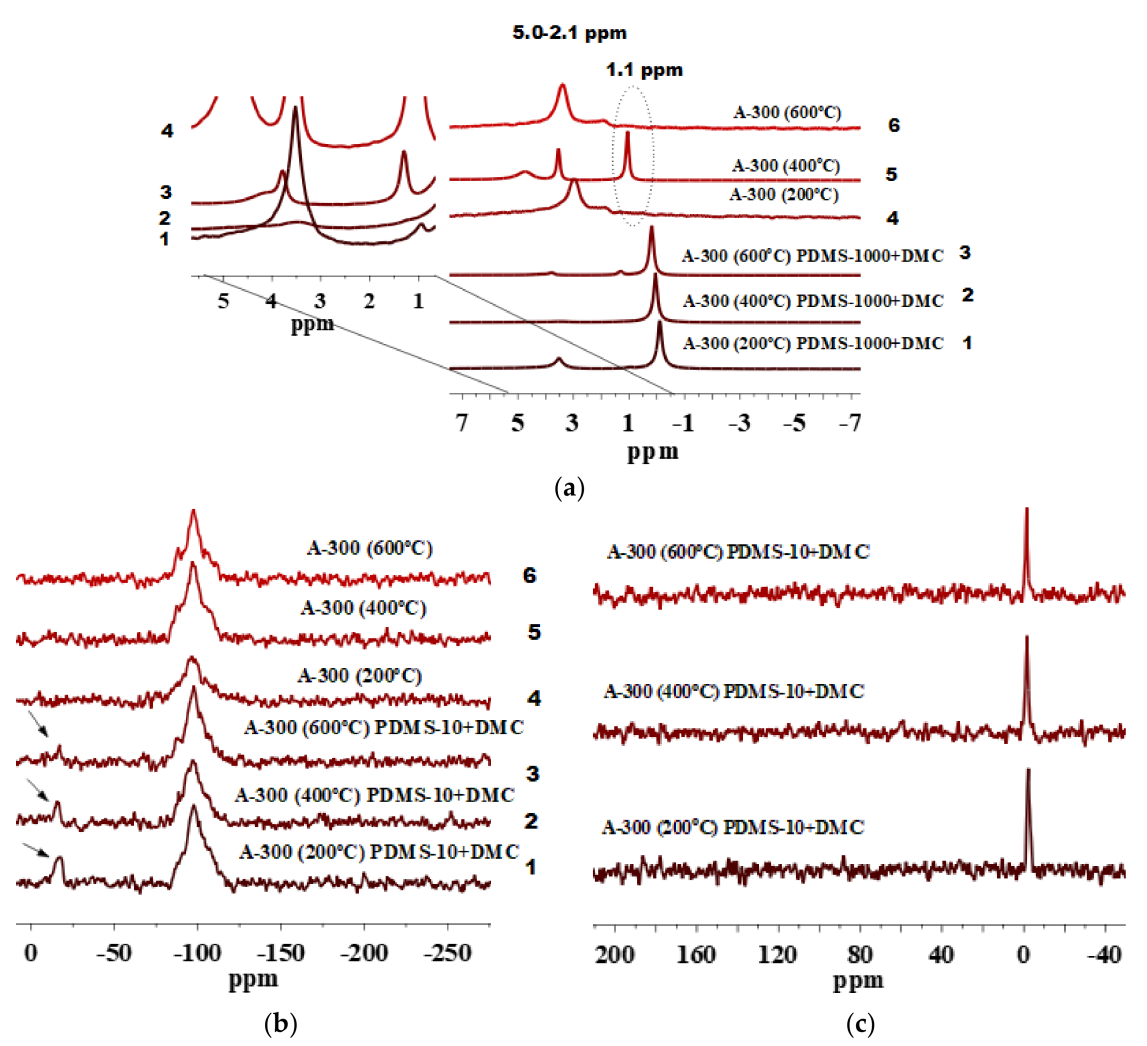
(**a**) ^1^H MAS NMR, (**b**) ^29^Si NMR, and (**c**) ^13^C CP/MAS NMR spectra of A-300 preheated at 200, 400, and 600 °C and modified by PDMS-10/DMC.

The ^1^H MAS NMR spectra of unmodified A-300 have the peaks at 2.1–5.0 ppm (Figure 8a, lines 4–6) of twin and vicinal silanols and intraparticle-bound water. Isolated silanols are not visible at 1.1 ppm for A-300 pretreated at 200 °C and 600 °C (Figure 8a, lines 4 and 6). However, signals of isolated silanols are visible for A-300 pretreated at 400 °C (Figure 8a, line 5). These effects can be explained by several temperature/morphology dependent processes of dehydrations similar to that affecting the SSA (Figure 1c). The signal at 0.0 ppm is present for all modified A-300 samples (Figure 8a, lines 1–3) that confirms the presence of the grafted PDMS species. Moreover, the ^13^C CP/MAS NMR spectra (Figure 8c) confirm this since the peak at −1.5 ppm is of relatively high intensity for all modified A-300 samples. The ^29^Si CP/MAS NMR spectra (Figure 8b) of these silica particles confirm the presence of PDMS functionalities characterized by the D^2^ peak of a moderate intensity for silica samples pretreated at 200 °C and 400 °C and of relatively lower intensity for silica samples pretreated at 600 °C. The difference in the D^2^ intensity could be explained by a low natural abundance of ^29^Si (4.7%) that requires extended acquisition times to obtain spectra with an acceptable sensitivity [64].

**Figure 9:**
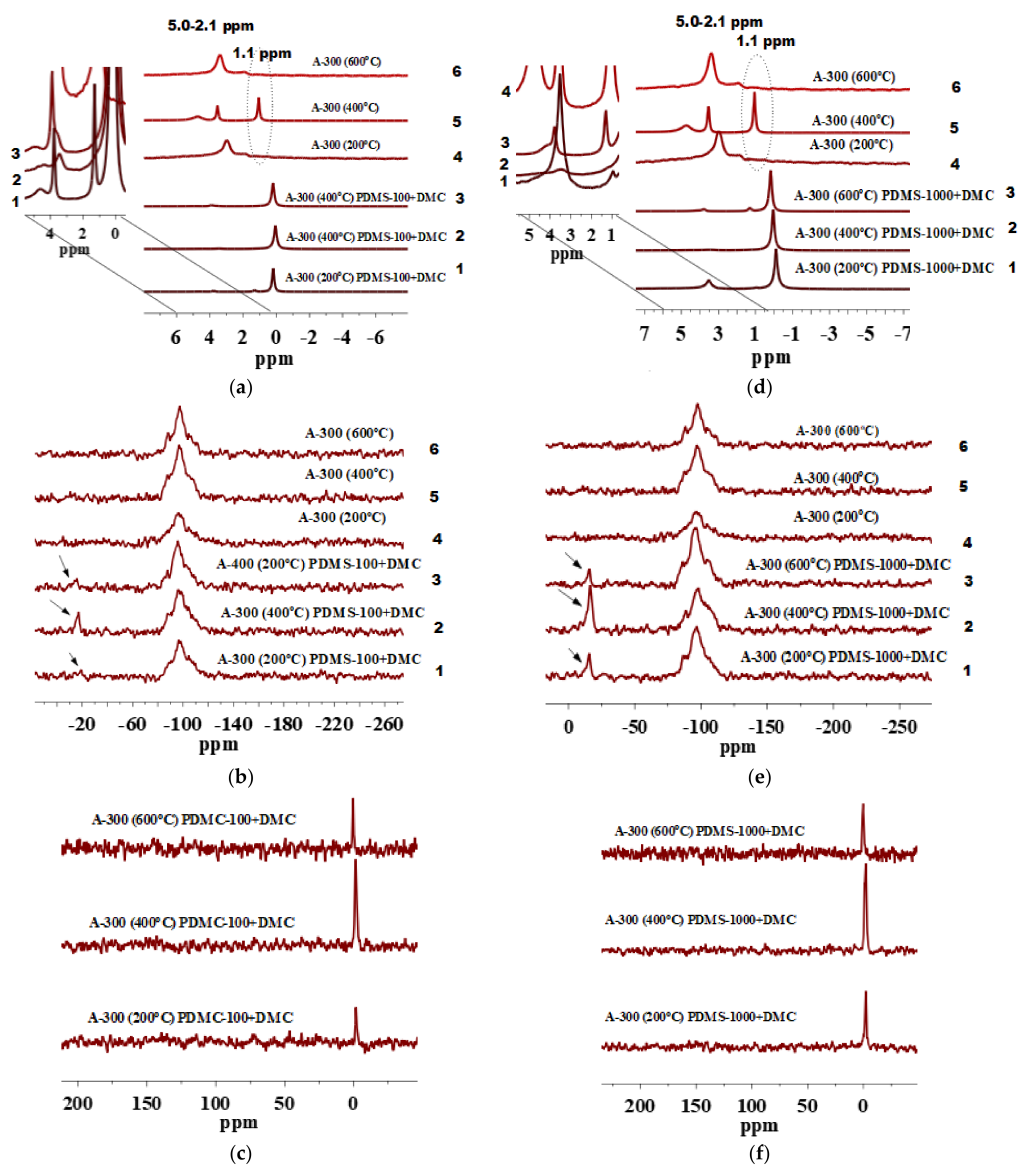
^1^H MAS NMR, ^29^Si CP/MAS NMR, and ^13^C CP/MAS NMR spectra of A-300 preheated at 200, 400, and 600 °C and modified by (**a**–**c**) PDMS-100/DMC and (**d**–**f**) PDMS-1000/DMC.

The ^1^H MAS NMR spectra of A-300 modified by PDMS-100/DMC (Figure 9a, lines 1–3) and PDMS-1000/DMC (Figure 9d, lines 1–3) confirm the presence of the grafted PDMS species since there is a peak at 0.0 ppm. The ^29^Si CP/MAS NMR spectra of both systems (Figure 9b,e, line 2) show that nanosilica A-300 samples pretreated at 400 °C and subsequently modified is characterized by a relatively high content of the grafted functionalities with D^2^ signals as compared with A-300 pretreated at 200 °C and 600 °C (Figure 9b,e, lines 1 and 3) and subsequently modified.

The grafting of PDMS species onto A-300 is also confirmed by the ^13^C CP/MAS NMR spectra (Figure 9c,f), peak at −1.5 ppm is present for all samples in both systems. However, the most intense peak is for the silica samples pretreated at 400 °C and modified with PDMS-100/DMC (Figure 9c) and silica samples pretreated at 400 °C and modified with PDMS-1000/DMC. This is in a good agreement with the ^29^Si CP/MAS NMR spectra (Figure 9b,e). This confirms that increasing polymer chain length used for silica modification does lead to a higher degree of grafting onto the same silica surface.

## 4. Conclusions

Nanosilica samples with different specific surface areas (SSA = 150, 200, and 300 m^2^/g), particle size distributions, and aggregation of nanoparticles in secondary structures were pretreated at different temperatures (200, 400, and 600 °C). Changes in the pretreatment temperature affects not only amounts of bound water and the content of surface hydroxyls, but also particle sizes and their aggregation features. All of these factors can affect the results of nanosilica particle functionalization by different polydimethylsiloxanes of 1.25–30 kDa in molecular weight (PDMS-10, PDMS-100, and PDMS-1000) in the presence of dimethyl carbonate (DMC) as a siloxane bond breakage agent. The functionalization features were analyzed with respect to the products of the chemical reactions between a modifier and a silica surface studied using solid-state NMR spectroscopy (H^1^ MAS, ^29^Si and ^13^C CP/MAS). It is found that the SSA value plays a significant role upon nanosilica modification by PDMS/DMC since the modification of various silica particles pretreated at the same temperature (e.g., 200 °C) and modified by the same modifier (e.g., PDMS-10/DMC) leads to different coverage degree with various fragments of PDMS. This can be explained by changes in the particulate morphology and texture of nanosilica particles with different SSA that affect the accessibility of surface sites, including silanols of different types.

It is found that for A-150 pretreated at 400 °C and then modified by PDMS-10/DMC and PDMS-1000/DMC, the grafting degree is higher than that for A-150 pretreated 200 and 600 °C and modified by the same modifiers. For A-200 pretreated at 400 °C and then modified by PDMS-10/DMC and PDMS-100/DMC, a relatively higher grafting degree is observed. For PDMS-1000/DMC-modified A-200, the pretreatment temperature plays a smaller role. For A-300, the highest degree of PDMS grafting is observed after preheating at 400 °C and then modified by PDMS-100/DMC and PDMS-1000/DMC. A-300 preheated at 200 °C or 600 °C and modified with the same modifiers shows a lower grafting degree of PDMS. For A-300 preheated at 200 °C and modified by PDMS-10/DMC, the grafting degree is higher than that after preheating at 400 °C and 600 °C and modified by PDMS-10/DMC. Thus, pretreatment of nanosilica samples at 400 °C gives better results than the pretreatment at lower (200 °C) or higher (600 °C) temperatures. This can be explained by overlapping of several effects caused by nanosilica preheating such as molecular and associative desorption of water from a surface and volume of nanoparticles, changes in nanoparticle aggregation, and textural characteristics. The sizes of the PDMS fragments formed upon the siloxane bond breaking by DMC, as well as the reaction rate upon DMC interactions with PDMS and silica surface, could depend on the PDMS length because of steric factors (the weight silica/PDMS and PDMS/DMC ratios were constant as 0.83:0.17 and 1:1, respectively). The detailed analysis of the ^1^H MAS, ^29^Si CP/MAS and ^13^C CP/MAS NMR spectra allows us to understand a set of the factors affecting the results of nanosilica functionalization by PDMS.

Thus, accurate description of a set of functional species at a surface of various PDMS/DMC-modified nanosilica samples using the solid-state NMR spectroscopy with ^1^H MAS, ^29^Si CP/MAS, and ^13^C CP/MAS allows us to obtain detail pictures of nanostructures at the interface. This is of importance to develop optimal synthetic protocols allowing tailoring surface sites and attached functionalities to synthesize functional nanostructured materials with optimal properties and characteristics.

## Figures and Tables

**Table 1:** Studied samples: pretreatment temperatures and types of modifiers.

Silica	Pretreatment temperature (°C)	PDMS (+DMC)
A-150	200, 400, 600	PDMS-10, PDMS-100, PDMS-1000
A-200	200, 400, 600	PDMS-10, PDMS-100, PDMS-1000
A-300	200, 400, 600	PDMS-10, PDMS-100, PDMS-1000

## Data Availability

The datasets supporting the conclusions of this work are included within the article. Any raw data generated and/or analyzed in the present study are available from the corresponding author on request.

## Sample Availability

Samples of the compounds are available from the authors.
